# Transcriptome Analysis of Targeted Mouse Mutations Reveals the Topography of Local Changes in Gene Expression

**DOI:** 10.1371/journal.pgen.1005691

**Published:** 2016-02-03

**Authors:** David B. West, Eric K. Engelhard, Michael Adkisson, A. J. Nava, Julia V. Kirov, Andreanna Cipollone, Brandon Willis, Jared Rapp, Pieter J. de Jong, Kent C. Lloyd

**Affiliations:** 1 Children’s Hospital Oakland Research Institute (CHORI), Oakland, California, United States of America; 2 Mouse Biology Program, University of California, Davis, California, United States of America; The Wellcome Trust Centre for Human Genetics, University of Oxford, UNITED KINGDOM

## Abstract

The unintended consequences of gene targeting in mouse models have not been thoroughly studied and a more systematic analysis is needed to understand the frequency and characteristics of off-target effects. Using RNA-seq, we evaluated targeted and neighboring gene expression in tissues from 44 homozygous mutants compared with C57BL/6N control mice. Two allele types were evaluated: 15 targeted trap mutations (TRAP); and 29 deletion alleles (DEL), usually a deletion between the translational start and the 3’ UTR. Both targeting strategies insert a bacterial beta-galactosidase reporter (LacZ) and a neomycin resistance selection cassette. Evaluating transcription of genes in +/- 500 kb of flanking DNA around the targeted gene, we found up-regulated genes more frequently around DEL compared with TRAP alleles, however the frequency of alleles with local down-regulated genes flanking DEL and TRAP targets was similar. Down-regulated genes around both DEL and TRAP targets were found at a higher frequency than expected from a genome-wide survey. However, only around DEL targets were up-regulated genes found with a significantly higher frequency compared with genome-wide sampling. Transcriptome analysis confirms targeting in 97% of DEL alleles, but in only 47% of TRAP alleles probably due to non-functional splice variants, and some splicing around the gene trap. Local effects on gene expression are likely due to a number of factors including compensatory regulation, loss or disruption of intragenic regulatory elements, the exogenous promoter in the neo selection cassette, removal of insulating DNA in the DEL mutants, and local silencing due to disruption of normal chromatin organization or presence of exogenous DNA. An understanding of local position effects is important for understanding and interpreting any phenotype attributed to targeted gene mutations, or to spontaneous indels.

## Author Summary

Insertion of foreign DNA into mammalian genomes, and the deletion of DNA, may have unintended consequences extending beyond the site of the mutation. In the mouse, the insertion of foreign DNA, including foreign regulatory DNA, combined with the deletion of part of the targeted gene, had striking effects on the regulation of neighboring genes. And this ectopic local gene dysregulation occurred at high frequency in regions of high gene density. These findings emphasize the importance of evaluating the local effects on gene regulation following spontaneous insertions and deletions, and after engineering mutations, in mammalian systems. Phenotypes associated with mutations in a specific gene may be partially or entirely due to effects on neighboring genes.

## Introduction

In mammalian systems, improvements of transgenic technologies have focused on methods to reduce the influence of surrounding DNA, also called position effects at the genomic insertion site, to ensure reliable and consistent expression of the transgene. To this end, strong reliable ubiquitously expressing promoters have been identified and engineered to provide appropriate and consistent transgene expression [1–3]. Exogenous DNA promoting gene silencing has been identified and removed from constructs [4], and insulating DNA has been utilized to reduce the influence of flanking DNA at the insertion site [5–6]. In addition, targeting vectors into safe havens with open chromatin [7] ensures that other genes are not disrupted and the targeting eliminates concatenation of the vector which may produce silencing [8]. The literature emphasizes a consideration of the impact of the local genome on the fidelity of the transgene [9–10] and generally doesn’t consider the effect of transgene integration on the local genome function. There are a few reports describing unintended position effects of either random integration or targeting events in mammals. And most of these reports relate to finding that a random integrant has disrupted either the regulatory domain [e.g., 11–12], or coding sequence of another gene. This lack of data may be a consequence of not looking.

In transgenic studies of commercial crop and animal species due to food safety concerns, investigators have evaluated unintended consequences and pleiotropic effects of transgene integration [for reviews see 13–14]. Since the majority of commercial plant and animal transgenics are not targeted, the approach has been to look at physiological parameters such as blood, nutritional value, e.g., carbohydrate composition of commercial crops, morphological changes by histology and transcriptome changes in multiple random integrants. And indeed they have been able to identify transgene specific vs. insertional position effects, primarily using transcriptome analysis [15]. Similarly, for viral delivery systems for human gene therapy, the unintended consequences of vector integration are of obvious concern for safety reasons [16–17].

The advent of expression profiling by the sequencing of cDNA libraries (RNA-seq) [18–20] enabled by NextGen sequencing has created an opportunity for using transcriptome analysis as a diagnostic tool to elaborate molecular mechanisms of disease [21], drug response [22], and response to toxins [23]. It is timely that we apply this technology to evaluate the consequence of the genomic insertion of targeted transgenic constructs.

The International Knockout Mouse Consortium (IKMC) is an international effort to create a resource of targeted gene-specific knockout C57BL/6N mouse embryonic stem cells for ~ 17,000 protein coding genes [24]. The next step in this process is to use these stem cells to produce live animals for cryopreservation and phenotyping and the International Mouse Phenotyping Consortium was formed to complete this initiative (IMPC) [25–26]. The NIH Common Fund supported Knockout Mouse Project (KOMP) is part of the IKMC and IMPC projects and has already produced targeted stem cells for ~ 7,500 genes, and approximately 2,500 of these have now been used to generate live mice. The KOMP project uses two separate targeting strategies [27–28]. In one approach (CHORI-Sanger-UC Davis, CSD) the construct is a targeted gene trap (TRAP) which can be converted to a conditional allele (see Methods). The TRAP is inserted into an intron 5’ to a critical exon and is designed not to disrupt any intronic regulatory elements. The vector construct is identical to that used by the European Conditional Mouse Mutagenesis Program (EUCOMM) [29]. In contrast, the Velocigene (VG) targeting strategy uses BAC recombineering to create a vector that deletes the entire open reading frame from the ATG of the targeted gene to the 3’ UTR creating a deletion allele (DEL). The inserted vectors for the CSD and VG alleles are very similar. Both targeting strategies leave behind a bacterial beta-galactosidase reporter (LacZ) and a neo selection cassette usually driven by a heterologous promoter. In order to evaluate local targeting effects, the use of the same targeting vector to target many different genes is ideal to evaluate the potential off-target effects since the vector remains unchanged but the local targeted environment is varied. In addition, we have two different targeting strategies which can be compared. In this report we evaluate the local effects of TRAP and DEL targeting events in C57BL/6N adult mice by using RNA-seq to characterize the expression of the targeted gene, and genes flanking the targeted gene, in tissues from 44 unique mouse lines.

## Results

### Expression of Neighboring Genes

**Fig 1:**
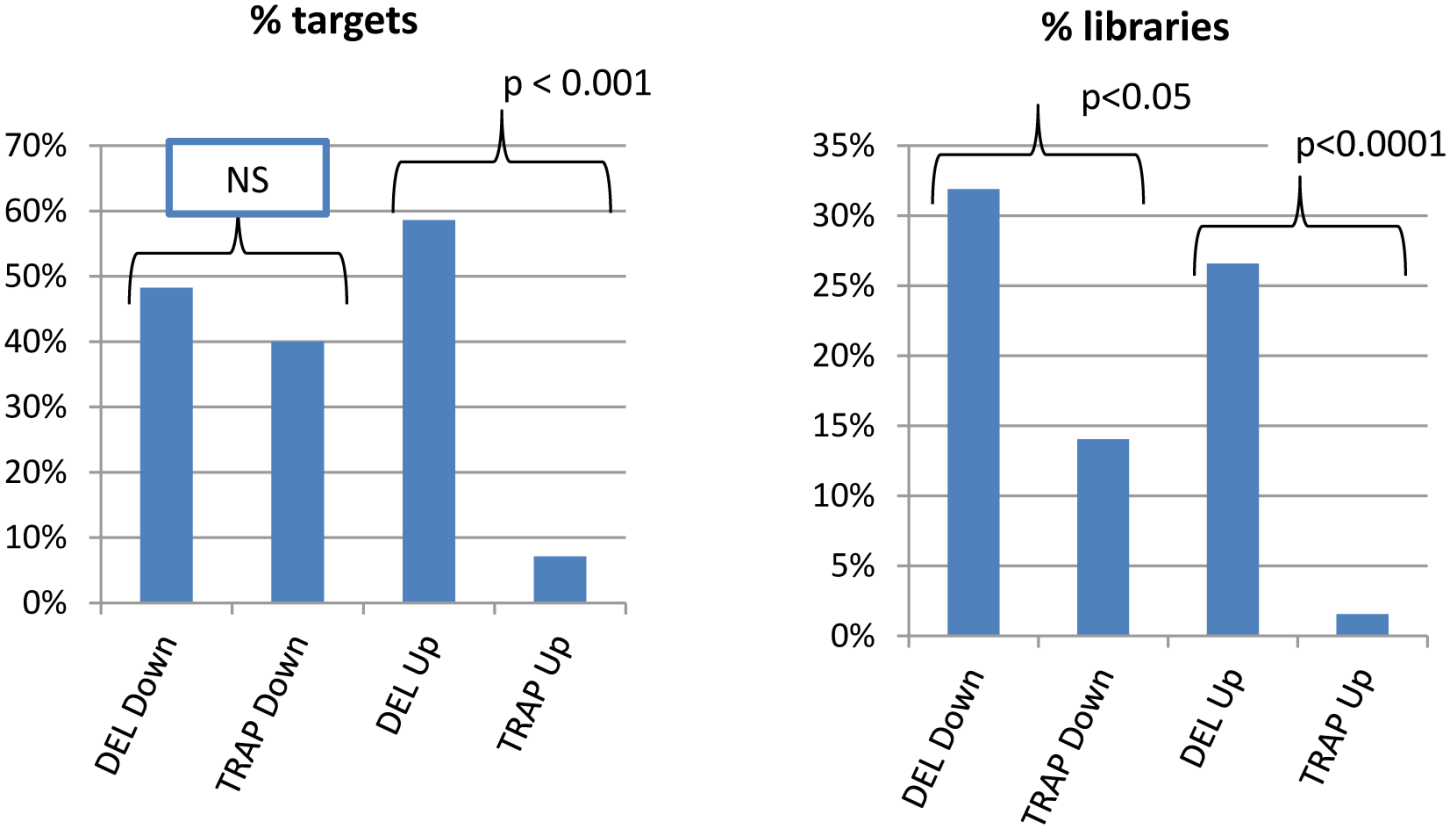
Percent of DEL and TRAP targets and libraries with one or more up- or down-regulated genes within 500kb of the target. A chi square statistic was used to compare the frequency of dysregulated genes between the two allele types. NS = not significant with p > 0.05.

We present here the changes in gene expression by RNA-seq for genes within +/- 500 kb of the center of the targeted gene. In S1 Table we present the data for this analysis including coordinates of all genes relative to the target gene for each sequenced library, the normalized read counts from library sequencing, the Log^2^ difference between homozygous (HOM) and wild-type (WT) gene expression, and the significance of the differences as determined by the DESeq2 statistical analysis (both the adjusted and unadjusted p value). Overall results are presented in Fig 1 for the gene trap (TRAP) mutants (15 targets and 64 sequenced mutant libraries), and for the deletion (DEL) mutants (29 targets and 94 sequenced libraries). Both TRAP and DEL targets had one or more down-regulated genes within 500kb of ~40–50% of the targeted genes. Whereas, 59% of the DEL targets, but only 7% of TRAP targets, had an up-regulated gene within 500kb. When the frequency of up- or down-regulated genes was expressed per library, similar differences were observed with more DEL libraries having both down- and up-regulated genes within 500kb compared with TRAP libraries (Fig 1).

**Fig 2:**
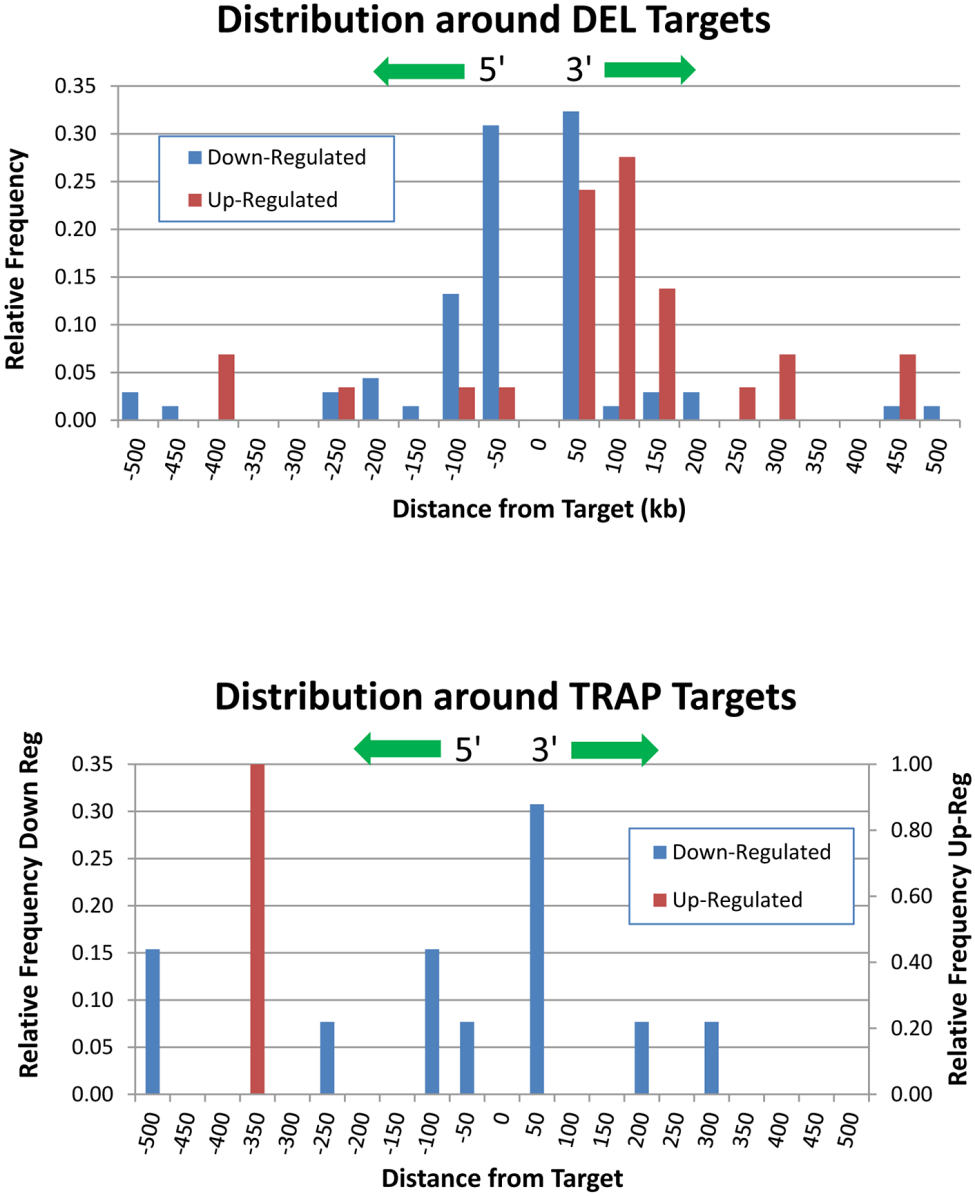
Spatial organization of up- and down-regulated genes flanking DEL and TRAP targets in HOM mutant mouse tissues. Genomic distances from the target are binned into 50 kb intervals with negative numbers for genes 5-prime of the target and positive numbers for genes 3-prime of the target. A total of 113 dysregulated genes are represented in these plots, not including the targeted gene.

There was a clear spatial organization of the up- and down-regulated genes flanking the DEL targets (Fig 2). Down-regulated genes flanking DEL targets were approximately equally distributed 5’ and 3’ of the target and the median distance from the target was 34kb. For up-regulated genes flanking DEL targets, there was a clear preponderance of up-regulated genes 3’ to the target (77.4%) vs. 5’ to the target (22.6%). The median distance of up-regulated genes 3’ to the target was 67kb, while the median distance of the up-regulated genes 5’ of the target was 321kb. For the TRAP targets, the down-regulated genes flanking TRAP alleles were approximately equally distributed 5’ and 3’ with the 5’ median distance of 224 kb, and the 3’ median distance from the target 8kb (Fig 2). There was only 1 up-regulated gene for one of the TRAP targets and it was located ~350kb 5’ to the target. There was no spatial organization of the genes with expression unaffected by the targeting event with approximately 5% of the genes (range 3.2–6.8%) found in each 50kb interval flanking the targets (derived from S1 Table).

We completed a genome wide assessment of the frequency of finding up-or down-regulated genes within 500kb of all genes down-regulated in the HOM mutant libraries. For this analysis all libraries were evaluated and a DESeq2 statistical significance of p < 0.05 was used as the criteria for identifying down-regulated genes. The overall genome-wide frequency for finding up-regulated and down-regulated genes within 500kb of genes down-regulated in the HOM libraries was 1.09% and 2.22%, respectively. This is much lower than the frequency of up- or down-regulated genes flanking the targeted genes. Using a chi-square statistic, we compared the actual frequency of up- and down-regulated genes flanking each of the mutant classes, with the genome-wide frequency (see Table 1). For DEL mutants, both up- and down-regulated genes were found flanking the targeted genes at a much higher frequency than the genome-wide frequency (p < 10^−6^). For TRAP mutants, the frequency of finding down-regulated genes was significantly greater than the genome-wide frequency (p = 0.016); while the frequency of up-regulated genes flanking TRAP mutations was not significantly different from the rate predicted by the genome-wide survey (p > 0.05).

**Table 1:** Frequency of up- and down-regulated genes in a genome wide analysis, flanking DEL mutations, and flanking TRAP mutations.

	Genome-Wide	DEL (n = 29)	TRAP (n = 15)
**% up-regulated**	1.1	48.3**	7.1^NS^
**% down-regulated**	2.2	58.6**	40.0^++^

NS not significant

Significantly different from genome-wide rate:

** p < 10–6;

^++^ p = 0.016

**Fig 3:**
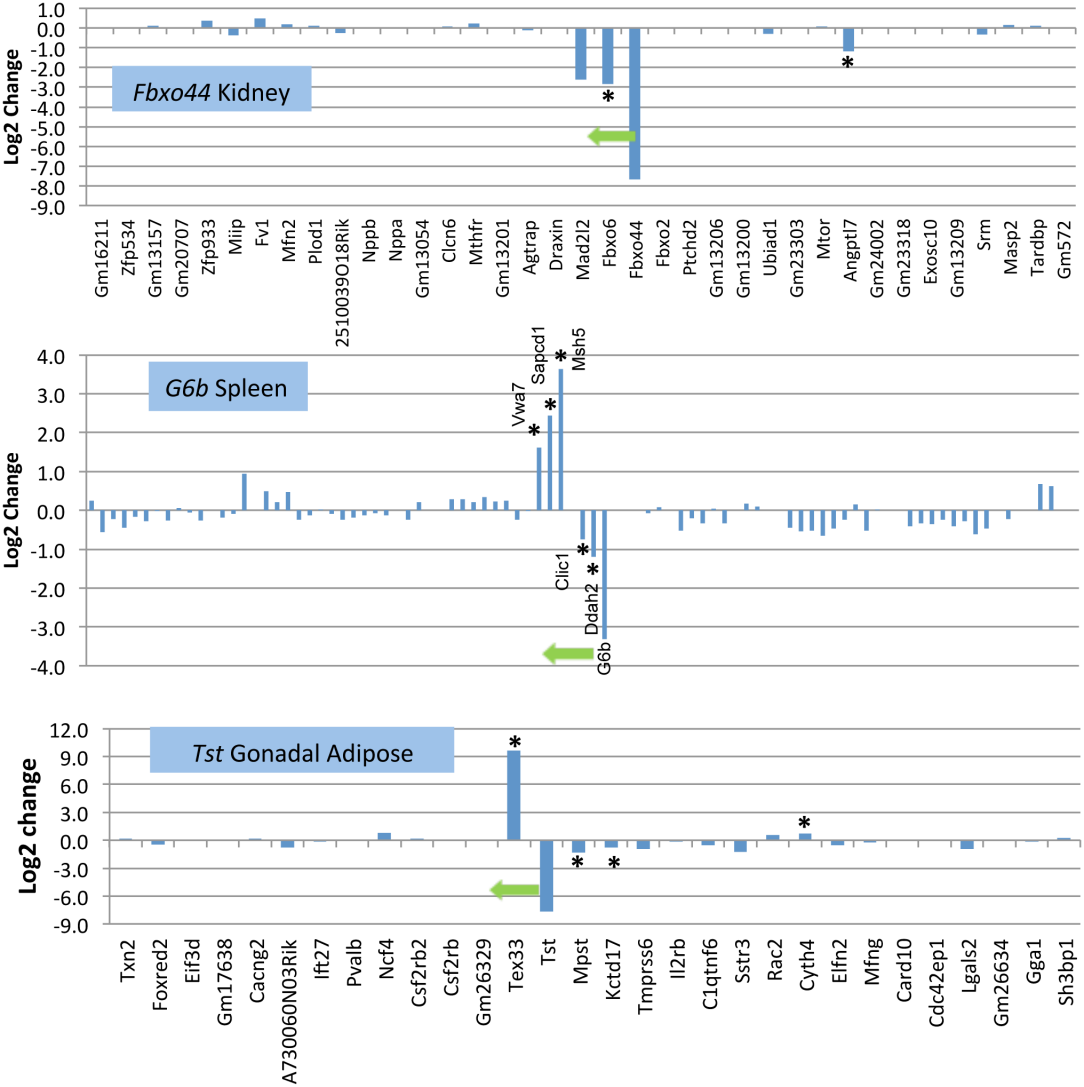
Regional effects of gene targeting in kidney of TRAP *Fbxo44* mutant (Top Panel), in spleen of DEL *G6b (AU023871)* mutant (Middle Panel), and in gonadal adipose tissue of DEL *Tst* mutant (Bottom Panel). The genes listed on the X-axis are all found in S1 Table for each targeted gene. In HOM *Fbxo44* kidney, flanking gene *Fbxo6* is significantly down-regulated relative to WT controls. In HOM *G6b* lung, two flanking genes are down-regulated, while three genes 3’ to *G6b* are up-regulated. In HOM *Tst* adipose tissue, gene *Tex33 3’* to *Tst* is highly up-regulated, while *Mpst1* 5’ and *Kctd17* are down-regulated. Gene symbols are listed in centromere to telomere order from left to right without consideration of distance or spacing. The total interval depicted in each graph is +/- 500 kb flanking the centrum of the targeted gene. Asterisks indicate a significant dysregulation by the DESeq2 statistic. Arrow indicates direction of targeted gene transcription.

Examples of local effects of targeting are provided for *Fbxo44*, a TRAP targeted gene (Fig 3, Top Panel), and two DEL targeted genes, *G6b* (Fig 3, Middle Panel), and *Tst* (Fig 3, Bottom Panel). In *Fbxo44* kidney, the flanking gene-family member *Fbxo6* was significantly down-regulated 2–4 fold and this was observed in 4 of 5 tissues (S1 Table). For the *G6b* HOM mutant spleen, three 3’ genes were significantly up-regulated, and 2 were down-regulated. In the *G6b* mutant, *Clic1* was significantly down-regulated in 3 of 4 tissues profiled, and the other genes showed similar trends as observed in *G6b* HOM spleen. (S1 Table). In *Tst* gonadal adipose, *Tex33* was highly up-regulated 3’ to *Tst*, while *Mpst* and *Kcdt17* were down-regulated 5’, and this same pattern was found in all four tissues although the changes do not reach DESeq2 significance levels in all tissues (S1 Table).

Of note are examples of local effects of the mutations when the targeted gene was not normally expressed in that tissue. Three examples of this were provided by mutations in the *Prkcdbp*, *G6b & Apof* genes (S1 Table). *Prkcdbp* was expressed at very low levels, but the neighboring gene *Cnga4* was significantly up-regulated in *Prkcdbp* mutant liver. *G6b* was not expressed in liver, but the *Clic1* gene was significantly up-regulated in *G6b* mutant liver as it was in all other mutant tissues examined. *Apof* was not expressed in the gastrocnemious muscle, but several genes in close proximity, including *Timeless* and *Pan2* were significantly dysregulated in *Apof* mutant gastrocnemious muscle.

Gene density was a predictor of finding genes up or down-regulated around the target. There were 27 mutants (21 DEL and 6 TRAP) with one or more up- or down-regulated genes within 500kb of the target. The average number of genes found within 500kb of these 27 mutant targets was 30.0 per target. For the 17 mutants without local effects (8 DEL and 9 TRAP), the average number of genes within 500kb was 18.1.

For the 44 targeted genes, based upon an analysis of transcription factor binding sites (TFBS), publicly available CHIPseq data, and coding sequence for miRNA, we conclude that there was a 6.7% frequency of the targeting event disrupting these regulatory elements in the 15 TRAP targets, while there was a 58.6% frequency for disrupting these intragenic regulatory elements in the 29 DEL targets. A chi-square test comparing the frequency of disruption of intragenic regulatory regions between DEL and TRAP alleles gave a p < 0.001.

### Expression of Targeted Gene

Comparing only total read counts between HOM targeted and WT mouse RNA-seq libraries, and relying upon the DESeq2 statistic, was not sufficient to confirm targeting for all 44 mutants. Expression of exons proximal to the gene trap, non-functional splicing around the gene trap, expression of the 3’ UTR or distal exons for deletion mutants, and reads mapping to intronic non-coding sequence, may have resulted in apparent failure of the targeting. In addition, with small numbers of replicates the DESeq2 statistic may not find significance despite large differences in read counts. Therefore, we used the criteria of a minimum of 100 reads in either the WT or HOM library, and relative reads in the HOM libraries of 20% of wildtype or greater to identify those mutants with possible failed targeting for additional follow-up. We followed-up by examining the patterns of read alignment to introns, exons, and untranslated regions of each gene.

**Fig 4:**
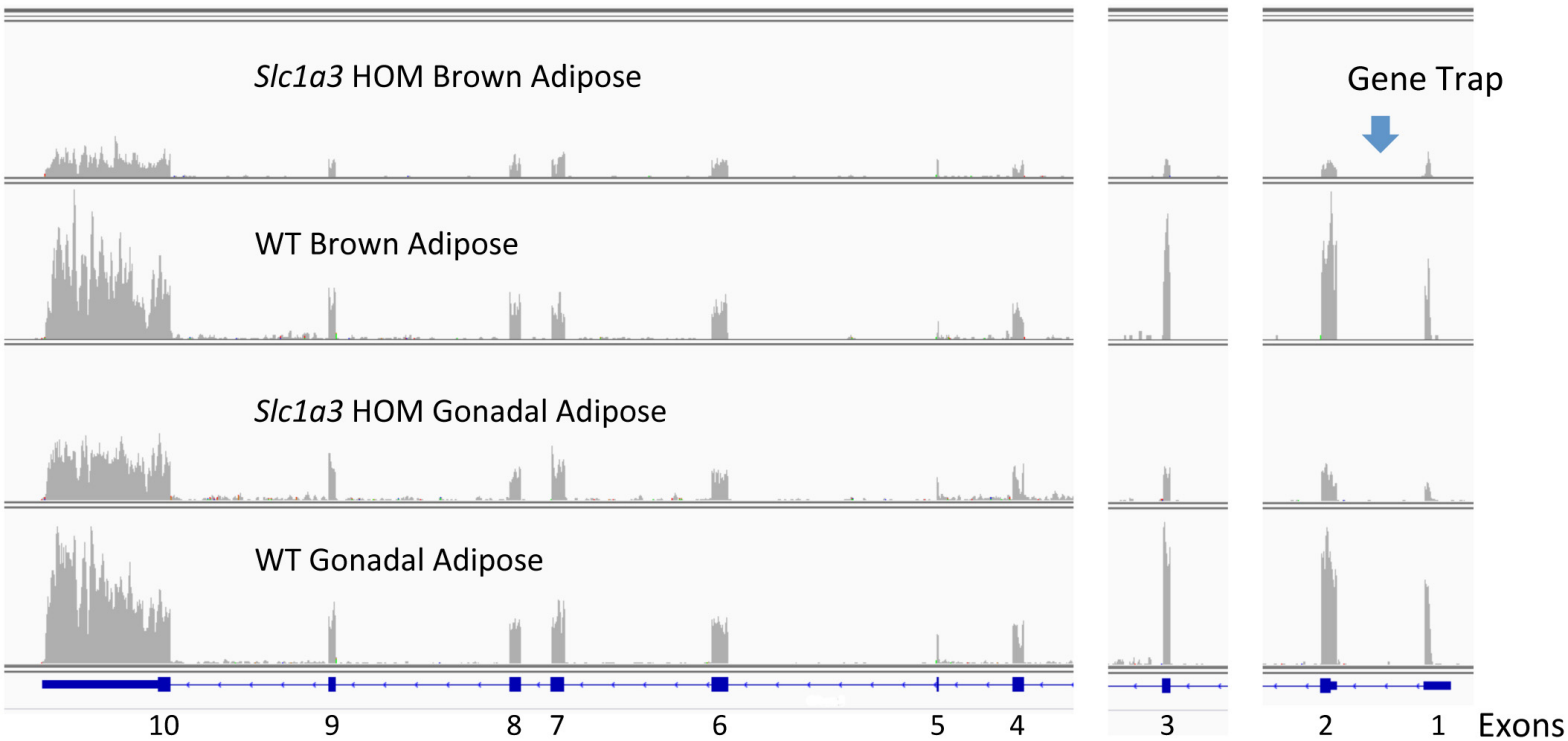
Reads for *Slc1a3* TRAP mutant and wildtype (WT) control interscapular brown adipose tissue and gonadal adipose mapping to *Slc1a3* genomic sequence. In both types of adipose tissue, reads from the mutant libraries map to each exon of the Slc1a3 gene. However, the number of reads is reduced relative to WT. This is a TRAP mutant with the gene trap cassette placed 5’ to exon 2. We found no reads for *Slc1a3* in libraries created from WT or HOM liver, and very low reads in abdominal muscle, therefore the graphs for those tissues are not presented. This visualization of Tophat2 mapping of RNA-seq read counts to the mm10 mouse reference genome sequence used the IGV reader. Exon segments are cut and pasted into position to show alignment of the reads against the genome reference sequence. At the bottom of each panel is a cartoon of the exons and introns with intronic arrows indicating the direction of transcription. Each panel is labeled with genotype and tissue. The read histograms, or pileups, show the numbers of reads at each nucleotide for each library. The Y-axis read count range of each graph for HOM and WT for the same tissue is adjusted to account for differences in total library reads. Therefore, the height of the bars for each HOM and WT tissue pair indicate the relative abundance of reads.

**Fig 5:**
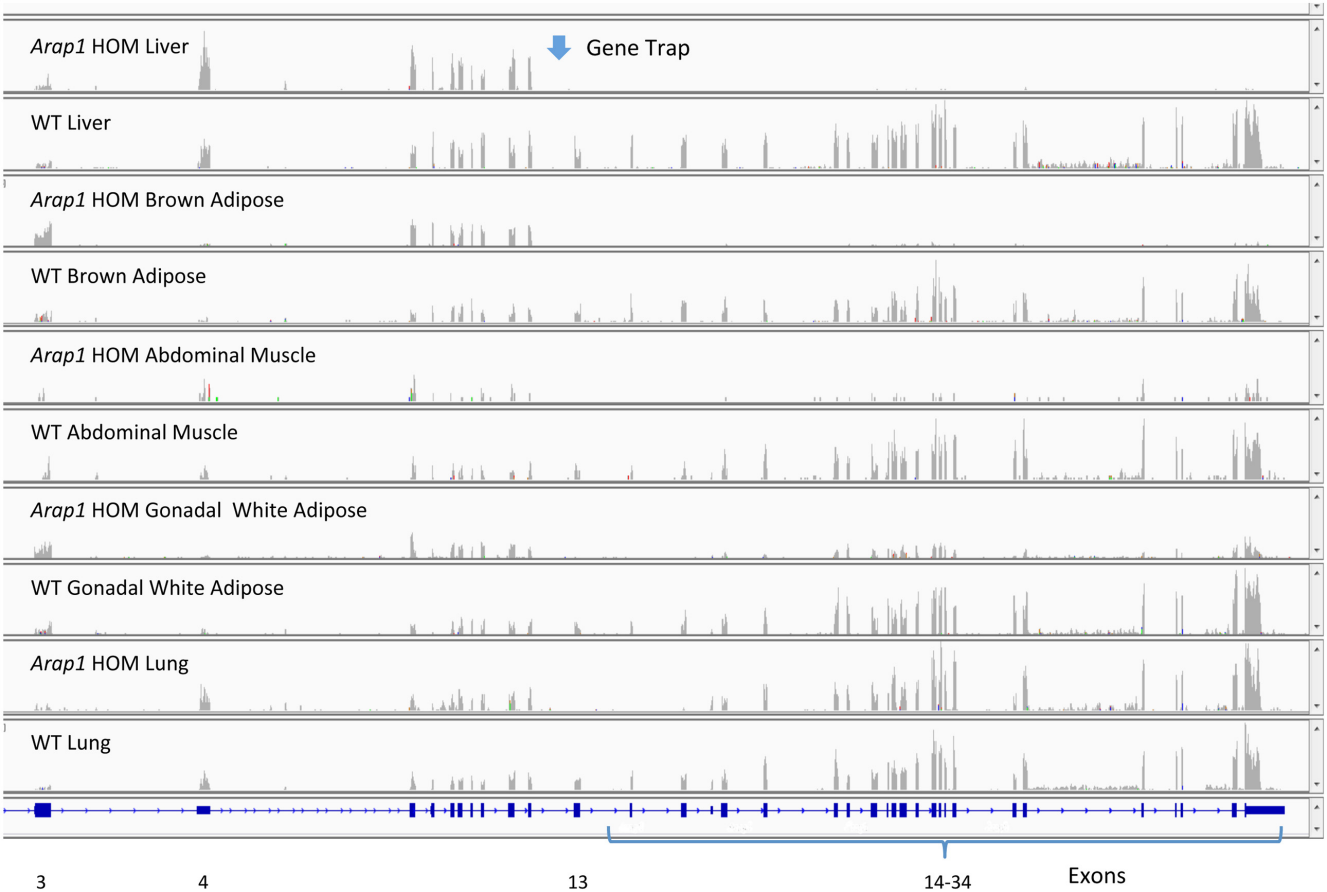
Reads for *Arap1* TRAP HOM and WT liver, brown adipose (interscapular), abdominal muscle, gonadal white adipose and lung mapping to *Arap1* genomic sequence. Exons 1 &2 are not shown because either very few or no reads mapped to those exons from the sequenced libraries. The TRAP vector targeting *Arap1* places the gene trap 5’ to exon 13. Liver and brown adipose tissue have no reads mapping 3’ to exon 12. However, for HOM abdominal muscle, gonadal adipose, and lung, after skipping exons 13, 14, 15 and 16, there are *Arap1* reads in the more distal exons, albeit at a lower count than those found in WT tissues.

Using the criteria described above, 8/15 TRAP mutants (47%) were evaluated further for targeting. Inspection of reads mapping to the mouse genome helped to confirm targeting, for example where reads mapped to exons proximal to the gene trap, or to untranslated regions. However, in some cases for TRAP alleles with reads mapping to the gene, the pattern of reads mapping to exons was identical to that found for WT controls, albeit at lower total read counts (e.g., Slc1a3, Fig 4). For TRAP mutant *Arap1*, the gene trap was complete in some tissues with no reads mapped 3’ to the gene trap in liver and brown adipose, while in other tissues the four exons 3’ to the *Arap1* TRAP were skipped and then reads were mapped to the exons in the distal 3’ half of the gene (Fig 5).

**Fig 6:**
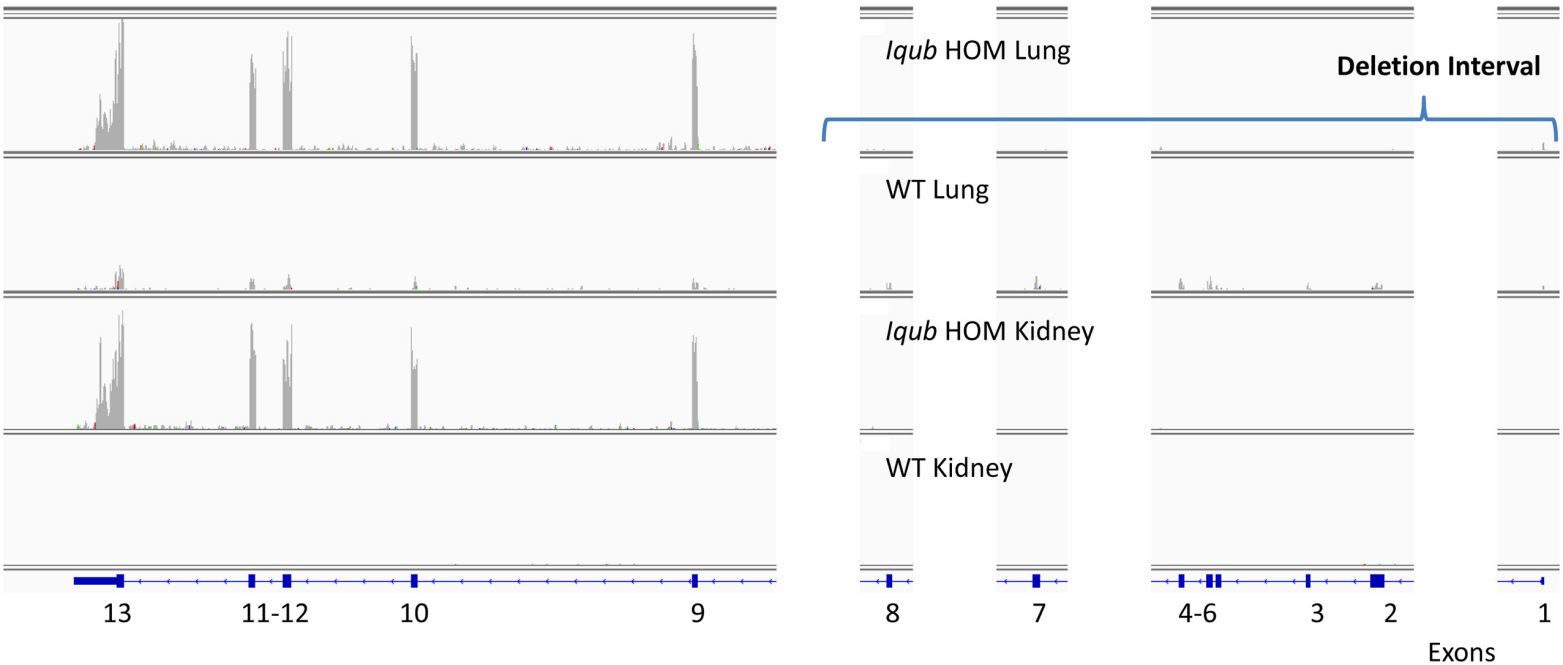
Reads for *Iqub* DEL HOM mutant and WT lung and kidney libraries mapping to *Iqub* genomic sequence. The *Iqub* gene is expressed at low levels in WT lung, and is not expressed in WT kidney. The expression in *Iqub* DEL HOM mutant lung and kidney is significantly increased compared with WT, but only for exons 9–13. This is consistent with targeting deletion of only exons 1–8 in the DEL mutant, and overexpression of the remaining exons in the mutant.

Six of twenty-nine HOM DEL mutants (21%) had at least 20% of WT reads mapped to the targeted gene. When we examined the alignment of reads for 4/6 of these mutants the patterns of read mapping confirmed that targeting was successful. Mutant *2700097O09Rik* had read counts mapped primarily to introns, *Iqub* had up-regulation of the distal exons and 3’ UTR with essentially no reads mapped to proximal exons in the mutant, *Klf14* had up-regulation of the 3’ UTR, and *Oxgr1* had low reads in the proximal exons but the distal exon had no reads. For *Iqub* and *Klf14*, the pattern of up-regulation of distal exons/3’UTR was likely a consequence of deletion of the proximal exons only and a compensatory over-expression of the remaining exons and 3’ UTR in those genes (e.g., *Iqub*, Fig 6). Only one DEL mutant line, *Adam26a*, had reads mapping to exons targeted for deletion (S1 Table and Short Read Archive Project #PRJNA280546). One gene, *Mtnr1b*, had no reads in either WT or HOM mutants mapping to the gene in any tissues and we could not conclude if it was correctly targeted based upon the RNA-seq data. Taking into account the location of the reads within each gene, we conclude that 97% of DEL alleles were correctly targeted.

## Discussion

### Local Effects of Targeting

Neighboring gene up- or down-regulation was found more frequently for DEL alleles than for TRAP alleles, and most of these differentially expressed genes were within 200 kb of the targeted gene. The frequency of down-regulated genes within +/- 500kb was significantly higher than expected from a genome wide survey for both DEL and TRAP mutants, whereas for up-regulated genes these only occurred above the genome wide expected frequency for DEL mutants and not for TRAP mutants. As would be expected, gene density around the target influenced the likelihood of local effects, since targets with local effects had approximately twice the number of genes in the 500kb flanking regions than genes with no local effects.

Some of the local gene differential expression around targeted genes may have been due to the co-regulation of gene clusters. Genes tend to cluster in euchromatin due to the requirement of an open DNA structure and regions of generally up- or down-regulated genes have been identified in mammalian genomes [30], but this doesn’t necessarily mean that the genes are co-regulated in response to developmental or cellular demands, or act in the same or related pathways. The presence of bidirectional promoters could also partly explain the co-regulation of flanking genes. Approximately 11% of human and mouse promoters are bidirectional [31, 32] and regulate expression of flanking genes. The function of these bidirectional promoters may be to maintain an open chromatic structure and to coordinate expression of gene networks [33]. Therefore, at least some of the local effects we found in this study could be compensatory feedback in response to loss of the gene product and/or the presence of bidirectional promoters. However, the frequency of local effects around the DEL targets was much higher than the 11% frequency of bidirectional promoters and suggests some other mechanisms were involved. In some cases, we found local effects in a specific tissue, identical to the local effects found in other tissues, even when the targeted gene was not normally expressed in that tissue, e.g., *Prkcdbp*, *Apof*, and *G6b* (S1 Table). And, we found that there was a unique pattern to the clustering of dysregulated genes around the DEL targets. Up-regulated genes were primarily 3’, with 77% 3’ of the targeted gene (Fig 2). Down-regulated genes were equally distributed 5’ and 3’ and were within 100kb of the targeted gene. The observations that gene dysregulation tended to cluster around the target even when the targeted gene was not expressed in that tissue, and the unique and different topography of up- and down-regulated genes, support the conclusion that some of these local effects were due to the targeting event and not a compensatory change due to the loss of targeted gene product.

We hypothesize that the local transcriptional dysregulation, was due to three mechanisms: 1) the targeting event disrupted regulatory and insulating elements within the targeted gene and this had cis-acting effects on flanking genes; 2) the exogenous promoter in the neo selection cassette acted on 3’ genes to either increase or decrease gene expression; and 3) insertion of ~ 5 kb of exogenous DNA, and elements in the vector, promoted gene silencing.

#### Disruption of intragenic regulatory sequences

There are many intragenic elements involved in the regulation of transcription found within introns and in the 5’ and 3’ UTRs of genes [34–35]. The intragenic elements include secondary promoters, enhancer, repressor and insulating elements, and sequence coding for miRNAs affecting stability of transcripts. There are examples of transgenes acting in cis to affect neighboring gene regulation due to the disruption of regulatory elements [e.g., 11–12, 36]. Disruption of cis-regulatory elements was more likely to occur in the DEL targeting strategy than in the TRAP strategy and may account for why local gene effects were more frequently found flanking DEL targets. Unintended disruption of miRNA sequence is of particular relevance since many miRNAs are found within introns of protein coding genes. Osokine et al. [37] reviewed the literature and found approximately 200 cases of unintended disruption of miRNA in mouse mutations and targeted mouse stem cell lines. In our analysis of the 44 targeted genes, we predicted that a higher proportion of DEL targeting events would disrupt intragenic regulatory elements than TRAP events (58% vs. 7%). This could explain the finding that up- and down-regulated genes were found with more frequency flanking DEL compared with TRAP targets.

In addition, enhancers and repressors found in complex promoters can act in cis on distant promoters through chromatin looping [38]. Deletion of the entire gene 3’ prime to the promoter in the DEL strategy would bring enhancer and repressor elements in the targeted gene promoter into closer proximity with downstream promoters and increase the opportunity for interactions. This is consistent with the preponderance of up-regulated genes in DEL targets found 3’ to the targeting event.

#### Heterologous promoter and neo cassette

There are a number of reports that targeted introduction of selectable marker genes disrupted the regulation of neighboring genes in the mouse [39–43]. For the majority of these reports, it is difficult to determine if the targeting disrupted a cis-acting regulatory domain, or if the presence of the neo selection cassette, including the promoter, affected neighboring gene regulation. These published examples used the *Pgk* promoter to drive neo expression. For Scacheri [43] it was clear that the presence of the heterologous promoter in the targeted gene was responsible for the lethality phenotype since the lethality was found in heterozygotes, and an aberrant transcript from the antisense strand of the *Pgk* promoter was expressed and possibly had dominant-negative effects. In the Ramirez-Solis report [42] targeting of mouse *Hoxb4* with a *Pgk*-Neo vector resulted in a phenotype that did not recapitulate the phenotype produced in a different mouse mutant caused by a point mutation.

Both the TRAP and the DEL vectors utilized a heterologous promoter and a neo selection cassette. However, these were different in a number of respects: including different promoters, *hACTB* and *hUBC*; and different polyA signals, SV40 and polyA from *mPgk*. Both the *hACTB* and *hUBC* are strong promoters [1–3, 44], the hUBC promoter has some bi-directionality [45], and both are active in embryonic stem cells [46]. One explanation for the difference in local gene effects between the TRAP and DEL mutants could be explained by differences in the selection cassette and not the targeting strategy.

It is possible that the TRAP or DEL vector linker regions, or neo coding sequence, contain cryptic splice donors and that the heterologous promoter drives expression of the neo cassette and it is then spliced around the polyA signal to exons in genes 3’ to the neo coding sequence. Meyers et al, [47] used *Pgk*-neo to target the mouse *Fgf8* and they reported aberrant splicing of the Fgf8 transcripts due to cryptic splice sites in the neo cDNA sequence. The TRAP and DEL neo sequences are identical except for nucleotides coding for three additional amino acids at the N-terminus, and the TRAP neo and the neo used by Meyers [47] are identical. Therefore, the cryptic splice acceptor/donors found in the neo by Meyers may also act in some of the TRAP and DEL vectors, leading to spurious reads mapping either to the targeted gene, or to exons in genes 3’ of the target.

#### Local silencing

In randomly integrated transgenes it is well established that repeated elements and concatenates of integrants [8, 48–49], and the presence of non-mammalian DNA especially of viral origin [50], are associated with silencing by histone modifications due to transacting factors and marked by cytosine CpG methylation. The vectors used for gene targeting, including the vectors used by both the TRAP and DEL strategies, generally avoid sequence that may promote silencing. However, both the LacZ and the neo resistance cDNA are of bacterial origin and the LacZ sequence specifically could be problematic. Several reports have described variegated expression of LacZ containing transgenes [51–52], suggesting silencing. Both the LacZ sequence and the neo-resistance gene sequence used in these constructs have high GC content. And one study reported that varying the CpG content of a LacZ reporter driven by the *hEF1α* promoter in transgenic mice significantly affected silencing of the promoter independent of insertion site. In that experiment, the silencing of LacZ expression was correlated with cytosine CpG methylation in the exogenous promoter, and was eliminated by reducing CpG content of the LacZ [53]. This suggests that the sequence of the LacZ cassette could activate methylation of local CpG islands in neighboring promoter regions. However, this has never been demonstrated in targeted transgenes inserting a LacZ reporter by homologous recombination.

### Confirmation of Targeting

For DEL alleles, RNA-seq confirmed targeting for 97% of the targeting events. However, for DEL mutants with a significant numbers of reads mapping to the targeted gene, it was necessary to examine the mapping distribution of the reads across the gene to reach a conclusion. For TRAP alleles, confirmation of targeting by using only total read counts from RNA-seq was ~50%. For the 50% of the TRAP targets with significant numbers of reads from RNA-seq mapping to the gene in HOM tissues, usually the reads mapped to all exons. This does not necessarily mean that the targeted gene was expressing a functional protein. There are examples of splicing around gene traps in mice in the literature [54–60]. Some of these examples of alternative splicing, or splicing around the gene trap sequence, produced functional protein and were hypomorphs [56, 58], and some produced message which was not translated [57]. This alternative splicing may be a consequence of a weak splice acceptor at the gene trap, or the presence of cryptic splice donors and acceptors within the neo cassette [47]. Splicing around the gene trap appears to be tissue specific. In the case of *Arap1* reported in this paper and described above with clear alternative splicing around the gene trap, this mouse gene has 19 splice variants, many of them protein coding, in the Ensembl database (ENSMUSG00000032812). Therefore, the Arap1 gene in particular may be transcriptionally organized to splice around the trap. In another more recent example of splicing around the gene trap, Hanstein et al. [61] evaluated the mRNA produced by a TRAP mutant for *Panx1*. They showed that intact mRNA was produced, although it was ~ 30% of that found in wild type mice, and that this TRAP mutant was a functional hypomorph which was adequate to recapitulate the phenotype in a true -/- null mutant. We also evaluated by RNA-seq the pattern of mRNA expression in this same TRAP mutant and also showed that *Panx1* mRNA was knocked down but not eliminated in the three tissues we sequenced (S1 Table). For any gene trap mutant, relying upon the presence or absence of RNA mapping to the exons of the targeted gene is not necessarily evidence that the gene is functionally expressed. If mRNA is detected and maps to the exons in an RNA-seq experiment, ultimately it is necessary to assess the amount of protein, and sequence the cDNA, in order to conclude if the splicing around the gene trap is likely to produce a functional protein.

### Conclusions

RNA-seq is a useful method to assess gene targeting efficiency and to determine if follow-up studies with mRNA sequencing and protein assays are needed to confirm the mutation. In addition, RNA-seq readily identifies up- and down-regulation of genes in close proximity to the target which may be compensatory or a consequence of the targeting strategy and vector. We report here that local effects of targeting occur in both TRAP and DEL targeted mutations but occur with higher frequency in DEL mutations. It is likely that, in some cases, these local effects on gene transcription surrounding the targeted site are a consequence of the presence of the LacZ/selection cassette in the TRAP and DEL mutants, and/or the deletion of the open reading frame in DEL mutants. Future work comparing local gene expression changes in TRAP and DEL mutants having intact targeting vectors, with those following Cre excision of the neo cassette, will help us determine if the neo cassette is responsible for the local gene expression changes. We do not know if, or how frequently, local gene expression changes due to the vector insertion, affect phenotypes in targeted mutants. However, one recent report using the identical TRAP allele targeting *Slc25a21* in the mouse revealed phenotypes that were a consequence of the down-regulation of a neighboring gene and not disruption of the targeted gene [62]. In some cases, up-regulation of a local gene may not have any physiological consequences unless the full pathway in which the protein product plays a role is active in that tissue. For genes flanking the target that are down-regulated, it is more likely that this would have a consequence in that tissue since the pathway is already active but expression is reduced in the mutant. When evaluating phenotypes in targeted mouse mutants, or in spontaneous indel mutations, one should consider the effect of the mutation on the expression and function of closely linked genes flanking the mutation.

## Materials and Methods

### Ethics Statement

This work was approved by the University of California Institutional Animal Care and Use Committee (UCD IACUC Protocol #17328) and was performed in accordance to the guidelines of the National Institutes of Health, Institute of Laboratory Animal Research and Guide for the Care and Use of Laboratory Animals.

### Gene List

The 44 genes for which homozygous (HOM) mutant tissues were evaluated by RNA-seq is: *2700097O09Rik*, *Aard*, *Adam26a*, *Adig*, *Apof*, *Arap1*, *Arl10*, *Atp6v1b1*, *Ccdc116*, *Ccl9*, *Cd248*, *Fastk*, *Fbxo44*, *G6b*, *Gkn2*, *Gnpda2*, *Gpr182*, *Il6ra*, *Inf2*, *Iqub*, *Jazf1*, *Kctd15*, *Klf14*, *Lancl2*, *Lpin3*, *Lrrc72*, *Lyplal1*, *Mtnr1b*, *Negr1*, *Oxgr1*, *Panx1*, *Plekha8*, *Ppapdc2*, *Prkcdbp*, *Rgcc*, *Sik1*, *Slc1a3*, *Slc7a13*, *Tex37*, *Tmem248*, *Tmem256*, *Tst*, *Ube2e2*, *Wtip*. Tissues examined in each mutant and allele type are listed in S2 Table.

### Mouse Production

Mutants were created with Knockout Mouse Project (KOMP; [24, 26, 63]), or EUCOMM [64], targeted embryonic stem cells derived from the C57BL/6N inbred mouse strain. The targeting strategies and cell lines have been described by Skarnes et al. [27] for the CSD and EUCOMM alleles, and by Valenzuela [28] for the Velocigene (VG) alleles. A description of each allele is available through the International Mouse Phenotyping Consortium (IMPC) [25]; or the Knockout Mouse Project (KOMP) Repository [65]. CSD/EUCOMM clones are gene trap (TRAP) alleles with the targeting vector expressing beta-galactosidase (LacZ) inserted 5’ to a critical proximal exon. The geometry of the CSD/EUCOMM allele was: splice acceptor, IRES, LacZ, SV40 polyA, *hACTB* promoter, neo, SV40 polyA. Two of the CSD/EUCOMM mutants we evaluated in this report did not have the standard *hACTB* promoter driving neo expression. One uses the *mPgk* promoter, and the other was promoterless and used the T2A self-cleaving strategy. VG targeting produced deletion alleles (DEL) that excised the entire gene between the translational start site and the 3’ UTR, leaving the LacZ at the ATG of the targeted gene. However, with very large genes only the 5’ exons/introns were removed. The geometry of the VG alleles was: LacZ cDNA, SV40 polyA signal, hUBC promoter, neo, Pkg polyA signal.

Mice were produced by injection of targeted stem cells into blastocysts. Chimeras were backcrossed to C57BL/6NTac or C57BL/6NCrl inbred mice and the resulting founding heterozygous mutants were expanded by additional backcrosses. Targeting was confirmed by long-range PCR [27] or the loss-of-allele assay [66] and zygosity for the targeted allele was confirmed by a qPCR assay specific for the LacZ sequence. Additional tests were completed as per Ryder et al. [67] to confirm vector integrity. HOM mice and control, wild type (WT) littermates were produced by breeding of heterozygous mice and weaned at approximately 21 days of age. Breeders and weaned animals were housed in an environmentally controlled animal facility on a 12:12 hour, light:dark cycle, with lights on at 07:00Hr. Mice were fed Harlan Teklad Global Rodent Diet #2918 with a composition of 18% protein and 6% fat. Food and water were available *ad libitum*.

Mice were euthanized at ~50-days of age by isoflurane anesthesia and thoracotomy. Tissues were rapidly removed and placed into RNAlater (Qiagen Inc), or were quick frozen in liquid nitrogen. Tissues removed for RNA extraction included duodenum, kidney, liver, lung, spleen, testis, cardiac ventricle, gastrocnemius or abdominal skeletal muscle, interscapular brown adipose tissue, epididymal gonadal adipose and stomach antrum.

### RNA-seq Analysis

RNA-seq analysis was completed on tissues from 15 mutant lines derived from CSD/EUCOMM clones (TRAP mutants) with an average of 4.2 tissues sequenced per mutant line, and on 29 mutant lines derived from VG clones (DEL mutants) with an average of 3.2 tissues sequenced per mutant line. For each HOM mutant mouse line, 4–5 male mice approximately 50 days of age were necropsied and tissues processed for RNA extraction. In addition, 5 age-matched WT mice were dissected and the tissues processed in parallel with the mutant mice. Tissues were homogenized with a bead mill in RNAzol-RT (Molecular Research Center, Inc.). DNA and protein was separated from RNA by water precipitation and the resulting aqueous phase was mixed with an equal volume of isopropanol. Total RNA was isolated using the RNeasy kit (Qiagen), including DNase treatment of the silica bound RNA to remove contaminating DNA. RNA quality was assessed by a Nucleic Acid Bioanalyzer 2100 (Agilent) and only RNA with an RNA Integrity Number > 8.0 was used for library production.

For RNA-seq analysis, total RNA from homozygous biological replicates was pooled for each mutant tissue after normalizing for concentration. cDNA libraries from mRNA were created using the TruSeq kit from Illumina (San Diego, CA). Single end sequencing with 50nt reads was performed on the Illumina HiSeq 2000 instrument using 4–5 multiplexed libraries per lane at the QB3 Sequencing Center at the University of California, Berkeley. The resulting sequence was parsed into individual libraries by barcode, and then preprocessed with the FastX Tool kit [68] to eliminate low quality and short reads using a minimum Phred score of 20 and a minimum read length of 18. Generally 95% of reads passed our quality trimming and the average number of trimmed reads was greater than 20M per library. We then aligned using the Burrows-Wheeler Aligner tool [69] against the Ensembl mouse NCBI m38 build, mm10 transcriptome and MiRBase miRNA database [70–71]. Approximately 90% of quality-trimmed reads mapped to the mouse transcriptome/miRNA reference sequences.

Comparisons between HOM mutant & WT reads were completed using the R Bioconductor package, DESeq2 [72]. RNAseq reads were mapped against the MM10 mouse reference genome using Tophat2 [73]. For verifying targeting, we used a criterion of normalized HOM targeted gene expression of less than 20% of WT gene expression. If the reads of the targeted gene in the HOM mutant exceeded this criterion, then we examined the distribution of HOM targeted gene reads mapped against the MM10 genome in order to determine the source of unexpected reads using the Integrated Genomic Viewer (IGV, v 2.3) from the Broad Institute [74–75]. For the regional effects analysis, for each library we compared read counts for HOM and WT libraries, for each gene and miRNA coding sequence within +/- 500 kb of the target. Up- or down regulated genes were defined as significantly different by DESeq2 (unadjusted p < 0.05). The unadjusted p value was used as a less stringent criterion since the study lacked power due to the low number of biological replicates. In addition to examining local effects, we determined the genome-wide frequency of detecting up- or down-regulated genes within 500kb of each gene in every HOM library that was significantly down-regulated relative to the wild-type reference library using the DESeq statistic with an unadjusted p < 0.05 criterion. A conservative 500kb interval flanking targeted genes was decided upon by a preliminary analysis of the data which showed that the majority of local effects occurred between 0 and 200 kb of the targeted genes (see results). All of the sequencing data generated for this study was deposited at the NCBI Short Read Archive (SRA; Project #PRJNA280546, KOMP Mouse Mutant Transcriptome Pilot).

### Bioinformatics Analysis of Regulatory Elements

To analyze the likelihood there were regulatory elements deleted by targeting, we used the Swissregulon Database [76–77]. Transcription factor binding sites (TFBS) were mapped to the intron disrupted by the selection cassette for TRAP targeting, or intronic and exonic TFBS disrupted by the DEL allele. In addition, we evaluated the CHIPseq tracks for mouse tissues on the UCSC Genome Browser [78–79]. Eleven different CHIPseq tracks were utilized to screen for Pol2, CTCF and p300 chromatin binding from 8 tissues. If the TRAP targeting vector was inserted into an intron between the promoter and 2 or more distal TFBS sites mapped with high confidence by Swissregulon, then that targeted event was scored as likely to have disrupted regulatory elements. Similarly, for the DEL targets, if two or more TFBS, along with corroborating data from CHIPseq tracks, were deleted by targeting, then that event was scored as likely to have disrupted regulatory elements. To compare the frequency of disruption of intragenic regulatory elements between TRAP and DEL targeting strategy, we used a chi-square goodness of fit test.

### Statistical Analyses

The default criterion for the R package DESeq2 was used to determine if a gene was up- or down-regulated in HOM mutants relative to WT controls with an unadjusted p < 0.05. For comparisons between DEL and TRAP mutants for the frequency of local up- or down-regulated genes, the presence of CpG or regulatory elements in the targeted promoter or disrupted introns, and for comparing the frequency of dysregulated genes within 500kb of the target, with the expected frequency based upon a genome-wide assessment, we used a chi-squared statistic.

## Supporting Information

S1 Table This table lists the RNA-seq normalized read counts for the target gene, and all genes flanking the target within +/- 500 kb of the centrum of the targeted gene. Data from all of the libraries sequenced are included in this table. Line = targeted gene. Synonym = synonym if available for the targeted gene. Tissue = tissue from which the cDNA library was produced. Chromosome = chromosome carrying the targeted gene. Gene = gene ID for all genes within +/- 500kb of the target. Start and End = the location of that gene on the chromosome, Centrum Distance = the distance of the gene from the center of the targeted gene in that library. Strand = the strand location for each gene ID. HOM = normalized readcounts from the mutant library. WT = wild type control normalized read counts. P value is the result of the DESeq2 statistic. HOM/WT = the ratio of the HOM to WT normalized read counts. Log2 = the log base 2 of the difference between the HOM and WT read counts.
(XLSX)

S2 Table This table provides the targeted gene ID for all of the mutants described in this report, along with the targeting strategy, and the tissues assayed. (XLSX)
